# Increasing Glucose 6-Phosphate Dehydrogenase Activity Restores Redox Balance in Vascular Endothelial Cells Exposed to High Glucose

**DOI:** 10.1371/journal.pone.0049128

**Published:** 2012-11-19

**Authors:** Zhaoyun Zhang, Zhihong Yang, Bo Zhu, Ji Hu, Chong Wee Liew, Yingyi Zhang, Jane A. Leopold, Diane E. Handy, Joseph Loscalzo, Robert C. Stanton

**Affiliations:** 1 Research Division, Joslin Diabetes Center, Harvard Medical School, Boston, Massachusetts, United States of America; 2 Division of Endocrinology and Metabolism, Huashan Hospital, Shanghai, China; 3 Brigham Woman's Hospital, Harvard Medical School, Boston, Massachusetts, United States of America; 4 Division of Endocrinology and Metabolism, Nanfang Hospital, Guangzhou, China; 5 Division of Endocrinology and Metabolism, 2nd Affiliated Hospital of Soochow University, Suzhou, China; Bristol Heart Institute, University of Bristol, United Kingdom

## Abstract

Previous studies have shown that high glucose increases reactive oxygen species (ROS) in endothelial cells that contributes to vascular dysfunction and atherosclerosis. Accumulation of ROS is due to dysregulated redox balance between ROS-producing systems and antioxidant systems. Previous research from our laboratory has shown that high glucose decreases the principal cellular reductant, NADPH by impairing the activity of glucose 6-phosphate dehydrogenase (G6PD). We and others also have shown that the high glucose-induced decrease in G6PD activity is mediated, at least in part, by cAMP-dependent protein kinase A (PKA). As both the major antioxidant enzymes and NADPH oxidase, a major source of ROS, use NADPH as substrate, we explored whether G6PD activity was a critical mediator of redox balance. We found that overexpression of G6PD by pAD-G6PD infection restored redox balance. Moreover inhibition of PKA decreased ROS accumulation and increased redox enzymes, while not altering the protein expression level of redox enzymes. Interestingly, high glucose stimulated an increase in NADPH oxidase (NOX) and colocalization of G6PD with NOX, which was inhibited by the PKA inhibitor. Lastly, inhibition of PKA ameliorated high glucose mediated increase in cell death and inhibition of cell growth. These studies illustrate that increasing G6PD activity restores redox balance in endothelial cells exposed to high glucose, which is a potentially important therapeutic target to protect ECs from the deleterious effects of high glucose.

## Introduction

Redox balance in cells is maintained by an interplay between processes that produce reactive oxygen species (ROS) and processes that eliminate ROS (antioxidants). Alterations in this highly regulated system may lead to cellular dysfunction or death. Many diseases have been shown to have alterations in the regulation of redox balance including diabetes mellitus [1]–[5]. Cell culture models of diabetes, animal models of diabetes, and humans with diabetes have increased ROS [2], [6]–[9]. Both increased production of ROS, as well as decreased antioxidant function have been shown to mediate the increased accumulation of cellular ROS [7].

Many research studies have demonstrated a central role for increased production of ROS in diabetes. The causes for increased ROS production are multifactorial, and include, but are not limited to, such important mechanisms as ROS production by mitochondria, by actions of advanced glycation end products, and by increased NADPH oxidase activity [2], [10], [11]. In addition, altered antioxidants also play a role in the elevated ROS levels in diabetes as follows.

The major antioxidant systems include the glutathione system, catalase, the superoxide dismutases (SOD) and the thioredoxin (Trx) system. Often not evaluated when the antioxidant function is studied is glucose 6-phosphate dehydrogenase (G6PD). Yet G6PD is the major source of the reductant NADPH upon which the entire antioxidant system relies. Glutathione reductase requires NADPH to regenerate reduced glutathione [12]. Catalase has an allosteric binding site for NADPH that maintains the enzyme in its most active tetrameric conformation and protects it against the toxicity of hydrogen peroxide [13]. SOD does not directly use NADPH but the action of SOD is to convert superoxide to hydrogen peroxide which then requires reduction either by the glutathione system or catalase to convert hydrogen peroxide to less toxic compounds [14]. Since catalase and the glutathione system depend on NADPH and that increased hydrogen peroxide will inhibit SOD [15], SOD function ultimately depends on NADPH. NADPH is also required for Trx reductase to convert the oxidized Trx to the reduced form [16], which plays a role in many important biological processes, including redox signaling. Hence these major antioxidant systems are dependent on the availability of NADPH that is principally produced by G6PD.

G6PD is the first and rate-limiting enzyme of the pentose phosphate pathway. In addition to maintaining the antioxidant system, NADPH is required for lipid biosynthesis, the cytochrome P450 system, nitric oxide synthesis, tetrahydrobiopterin synthesis, HMG CoA reductase, and NADPH oxidase (NOX). Work from our laboratory and others has shown that G6PD is the principle source of NADPH for many of these processes [17]–[22]. In addition, we and others have determined that high glucose stimulates protein kinase A (PKA) that, at least in part, causes the decrease in G6PD and NADPH. In this study, we hypothesized that the high glucose-induced decrease of G6PD activity is a major cause of the redox imbalance in endothelial cells and that increasing G6PD activity will rescue the ECs from the deleterious effects of high glucose. The results reported here show that increasing G6PD activity by two different methods (overexpression of G6PD and inhibition of PKA) restores redox balance in ECs exposed to high glucose.

## Results

### High glucose decreased antioxidant systems in endothelial cells

**Figure 1:**
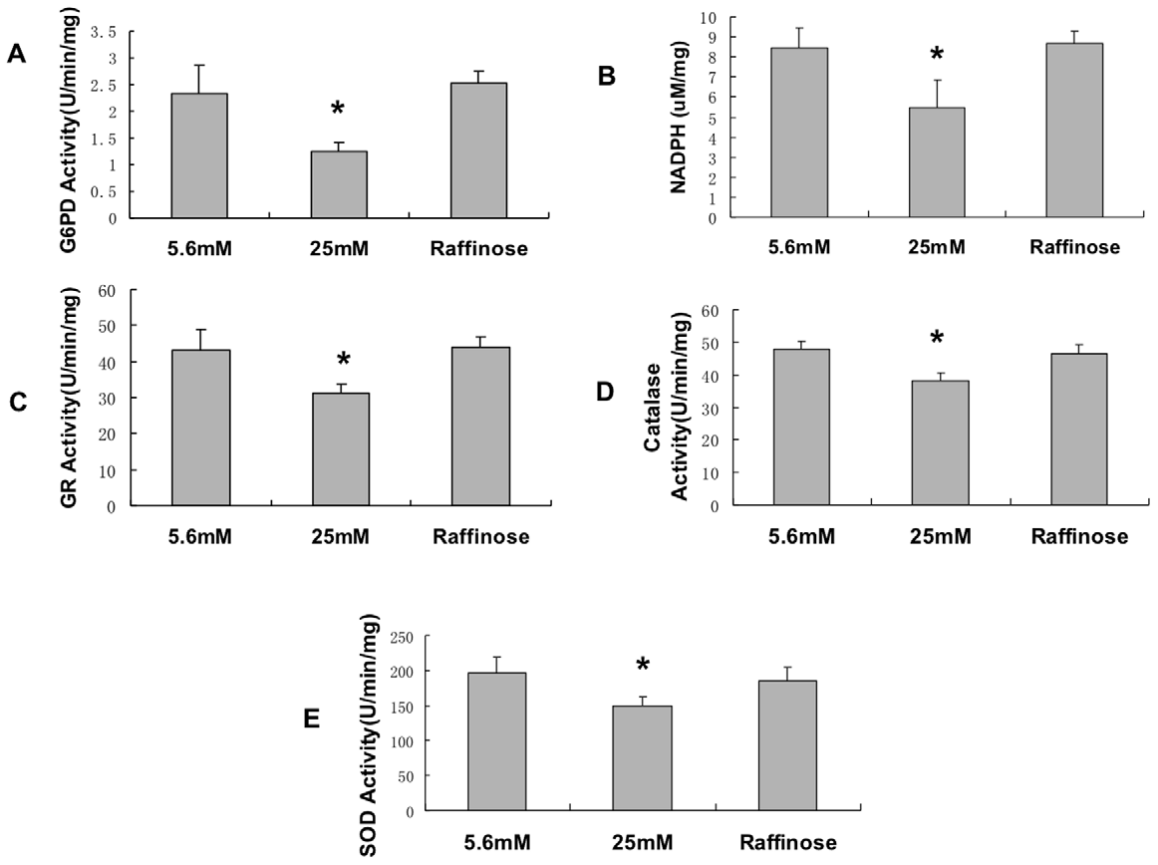
High glucose decreases antioxidant activities in endothelial cells. Bovine aortic endothelial cells were grown in DMEM (5.6 mM glucose) with 10% serum until 80% confluent and then switched to 0.5% serum plus 5.6 mM or 25 mM glucose for 72 hours. Raffinose was used as an osmolarity control. Measurements were performed as described in Methods. High glucose causes a decrease in multiple antioxidant enzymes. A: G6PD activity. B: NADPH level. C: Glutathione reductase activity. D: Catalase activity. E: Superoxide dismutase (SOD) activity. *, p<0.05 compared with 5.6 mM and raffinose conditions. Data were normalized by protein concentration and expressed as mean ±S.E in all figures. n  = 5. The n's in all figures reflect separate experiments not separate plates of cells.

**Figure 2:**
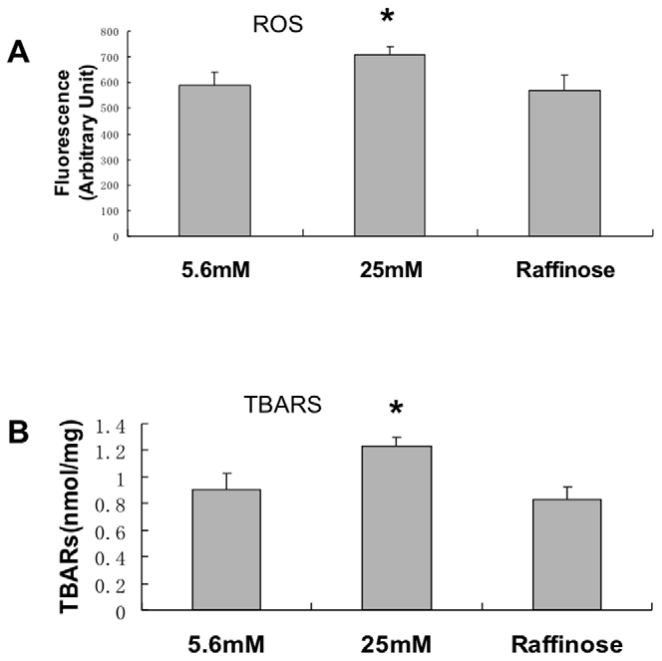
High glucose increased ROS (reactive oxygen species) generation in endothelial cells. Cells were prepared as in Figure 1. High glucose caused increased RO and increased TBARS. A: ROS level was measured with H_2_DCFDA (see Methods). B: TBARs level was measured as described in Methods. *, p<0.05 compared with 5.6 mM and raffinose conditions. n = 6.

Initially we verified that high glucose decreased G6PD activity in this experimental system as previously described. In Figure 1, bovine aortic endothelial cells were exposed to 5.6 mM or 25 mM glucose for 72 hours. As observed previously, high glucose caused a decrease in G6PD activity (Figure 1A) and NADPH level (Figure 1B). Interestingly high glucose led to significantly decreased activities in glutathione reductase (GR), catalase, and superoxide dismutase (Figure 1C, 1D, and 1E). High glucose also caused an increase in ROS (Figure 2A). To confirm that the cellular milieu was indeed in a state of redox imbalance favoring increased ROS, it was determined that there was an increase in oxidized lipids as measured by thiobarbituric reactive substances (Figure 2B). Taken together, these results show that high glucose causes redox imbalance in ECs that is associated with impaired operation of antioxidant systems.

### Overexpression of G6PD improved antioxidant enzyme activity and reduced ROS levels in endothelial cells

**Figure 3:**
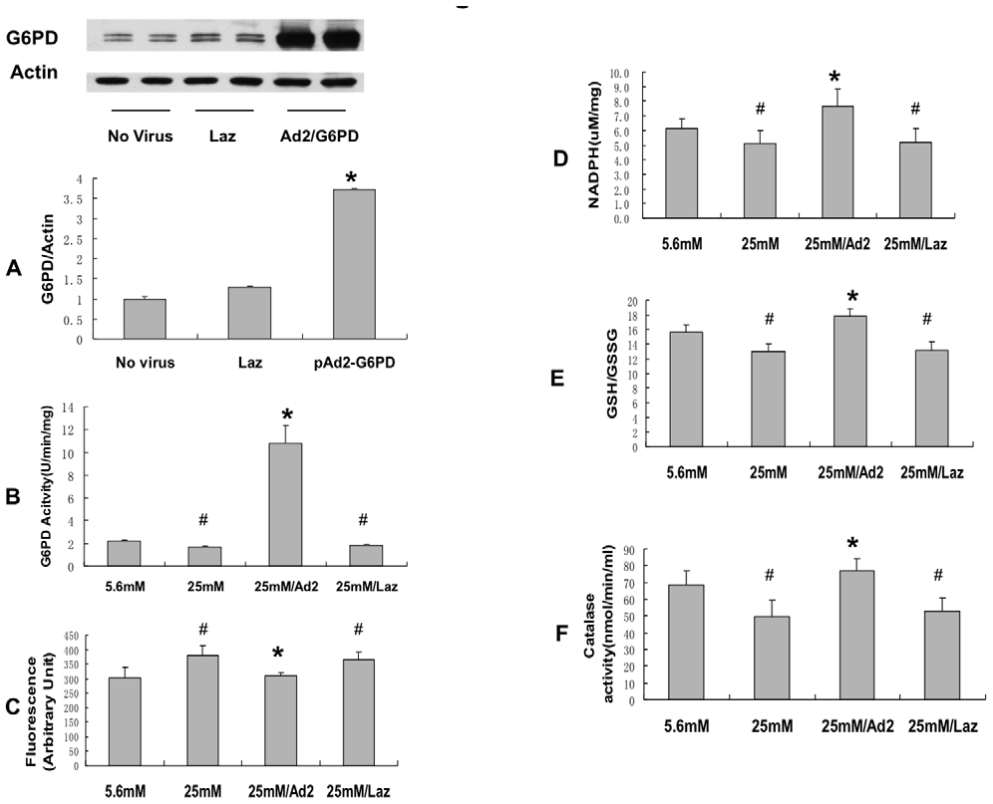
Overexpression of G6PD rescued the high glucose-indueed decrease in the antioxidant enzymes and reduced ROS level in endothelial cells. Adenovirus vector inserted with human G6PD cDNA was constructed and purified as described in the Methods. Endothelial cells were infected with either Ad2-G6PD (MOI: 5) or empty vector control (Laz). A: G6PD protein was significantly increased with adenovirus infection in endothelial cells exposed to high glucose. Overexpression of G6PD led to the following changes in cells exposed to high glucose as compared to cells exposed to high glucose with wild type G6PD activity: B: G6PD activity was increased. C: ROS level was decreased. D: NADPH level was increased. E: GSH/GSSG level was increased. F: Catalase activity was increased. *, p<0.05 compared with 25 mM conditions. #, p<0.05 compared with 5.6 mM condition. n = 8.

Cells were infected with either an empty adenovirus or an adenoviral vector containing human G6PD (pAd-G6PD). pAd-G6PD infection resulted in an approximate 5-fold increase in G6PD expression and activity (Figures 3A and 3B) and about a 60% increase in NADPH level (Figure 3D). Overexpression of G6PD caused both a decrease in ROS (Figure 3C) and an increase in the GSH/GSSG ratio reflecting an overall decrease in the intracellular ROS level (Figure 3E). Interestingly, Figure 3F shows that overexpression of G6PD also rescued the high glucose-induced decrease in catalase activity. Overexpression of G6PD caused no change in catalase protein level (Figure S1). As catalase has a critical allosteric binding site for NADPH that maintains the enzyme in its active conformation [13], it is possible that overexpression of G6PD directly increased catalase activity by providing NADPH for the allosteric binding site. Overexpression of G6PD also led to a trend to rescuing of glutathione reductase (GR) and superoxide dismutase (SOD) activity that did not quite reach statistical significance (data not shown) and no change in GR or SOD protein levels (Figure S2 and S3). Overall these results suggest that the decrease in the antioxidant systems is in significant part due to the high glucose-mediated decrease in NADPH.

### Pharmacologic Inhibition of protein kinase A rescued the high glucose-induced decrease in antioxidant enzymes

**Figure 4:**
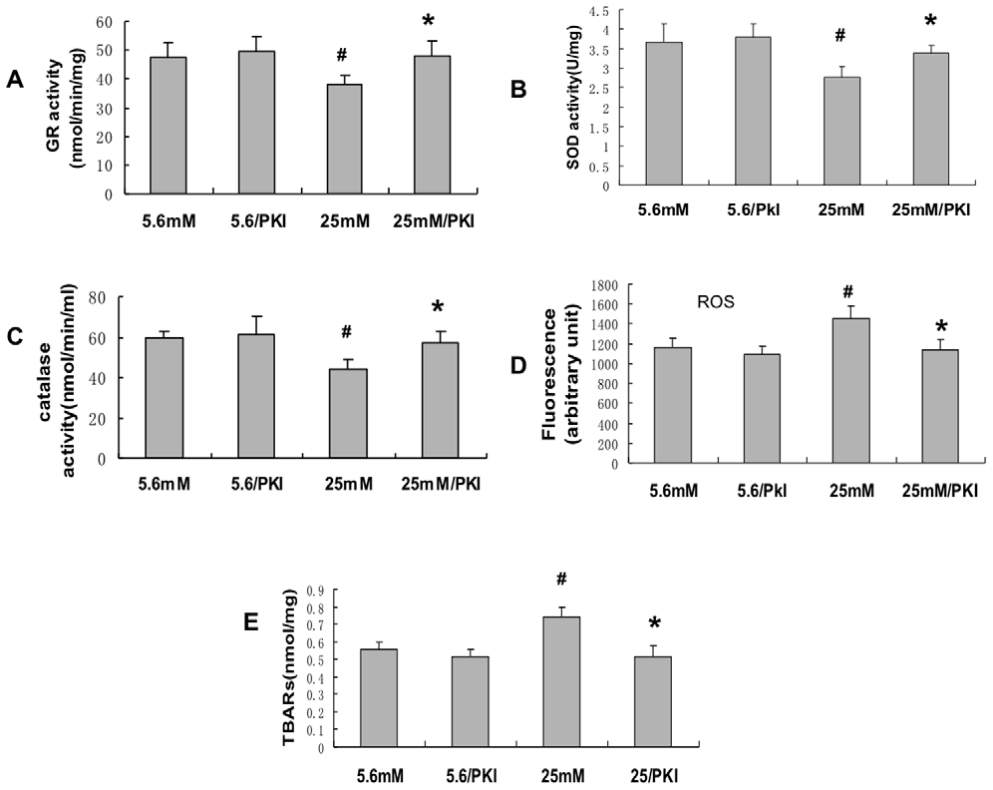
Pharmacologiic Inhibition of protein kinase A improved antioxidant activities in endothelial cells. High glucose increases cAMP, at least in part by activation of adenylate cyclase, which leads to activation of PKA (see text) and subsequent inhibition of G6PD. To inhibit PKA, endothelial cells were treated with a specific cell-permeable PKA inhibitor 14–22 amide (10 µmol/l) for the last 24 hours. Addition of PKI to cells exposed to high glucose led to: A: Glutathione reductase activity increase. B: SOD activity increase. C: Catalase activity increase. D: ROS level decrease. E: TBARs level decrease. *, p<0.05 compared with 25 mM condition. #, p<0.05 compared with 5.6 mM condition. n = 8.

Work from our laboratory and others has shown that high glucose stimulates an increase in cAMP and protein kinase A, which mediates, in significant part, the decrease in G6PD activity and NADPH level [9], [23]. Thus if increased PKA mediates the decrease in G6PD activity and NADPH level and in turn, these changes cause the high glucose-mediated decrease in the antioxidant enzyme activities of GR, catalase, and SOD as suggested in figure 3, then inhibition of PKA should rescue the glucose-induced increase in these enzymes. Using the cell-permeable PKA inhibitor 14–22 amide (PKI, Figure 4 illustrates that PKI rescued the high glucose-stimulated decrease in GR (Figure 4A), SOD (Figure 4B) and catalase (Figure 4C). Figure 4D also demonstrates that inhibition of PKA led to a decrease in ROS and Figure 4E shows that inhibition of PKA decreased TBARS, as well. Taken together, these results suggest that high glucose stimulates PKA leading to a decrease in G6PD and NADPH level and subsequent decrease function of GR, catalase, and SOD.

### siRNA oligonucleotide targeted to protein kinase A rescued the high glucose-induced decrease in antioxidant enzymes

**Figure 5:**
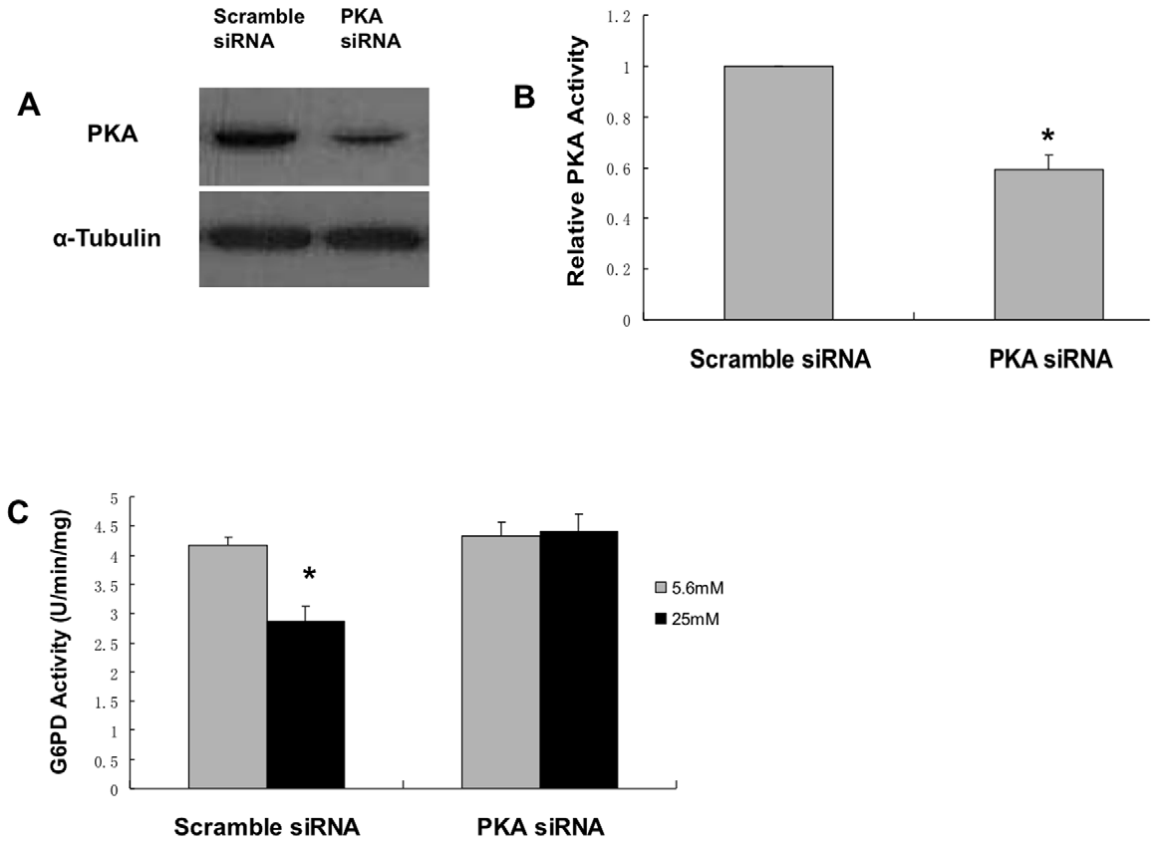
siRNA oligonucleotide specific for PKA causes decreased expression and activity of PKA and ameliorated the high glucose mediated decrease of G6PD activity. BAEC were transfected with duplex siRNA targeted against PKA (PKA siRNA) or a random sequence (scrambled siRNA). 48 h after transfection, cells were harvested and lysed, PKA activity was measured and protein levels were analyzed in immunoblots probed with a PKA antibody or tubulin antibody, as shown. *, p<0.05 compared with scramble siRNA. Figures A and B show that siRNA led to decreased expression and decreased activity of PKA. In figure 5C, BAEC were transfected with duplex siRNA targeted against PKA (PKA siRNA) or a random sequence (scramble siRNA), after 24 hours, medium was switched to DMEM with 1% serum plus 5.6 mM glucose or 25 mM glucose for 72 hours. G6PD measurements were performed as described in Methods. *, p<0.05 compared with 5.6 mM condition. n = 6.

**Figure 6:**
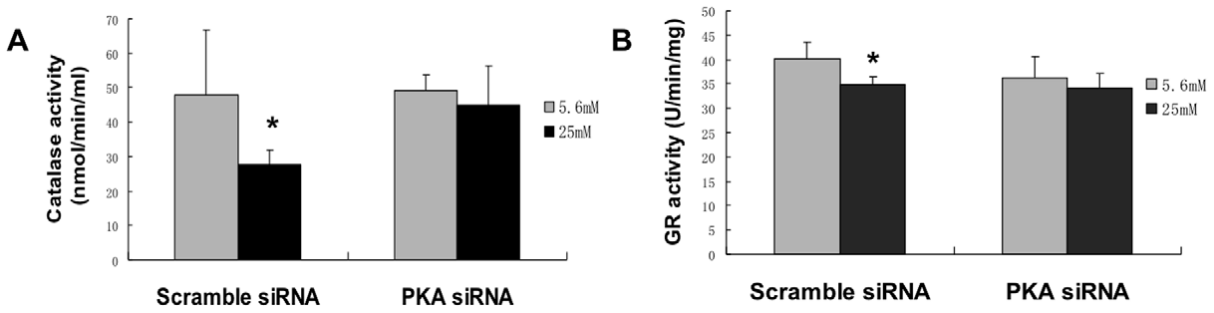
Inhibition of PKA by siRNA improved antioxidant activities in endothelial cells. Cells were transfected with siRNA and then treated with 5.6 mM glucose or 25 mM glucose for 72 hours: A: High glucose mediated decrease in catalase activity is prevented by siRNA. B: High glucose mediated decrease in glutathione reductase activity is prevented by siRNA. *, p<0.05 compared with 5.6 mM condition. n = 6.

To verify that the pharmacologic inhibition of PKA was specific for PKA, a small interfering RNA oligonucleotide was used as described in the methods. Figure 5A reveals that the siRNA oligonucleotide significantly decreased the expression of PKA and Figure 5B illustrates that PKA activity was similarly decreased. Figure 5C demonstrates that the high glucose mediated decrease in G6PD activity is ameliorated when the cells are transfected with siRNA for PKA showing that PKA is a significant inhibitor of G6PD under high glucose conditions. Next, the effect of siRNA on the enzymes catalase and glutathione reductase was studied. Figure 6 illustrates that siRNA rescued the high glucose induced decrease in catalase and glutathione reductase.

### Inhibition of protein kinase A by siRNA enhanced cell growth and decreases cell death

**Figure 7:**
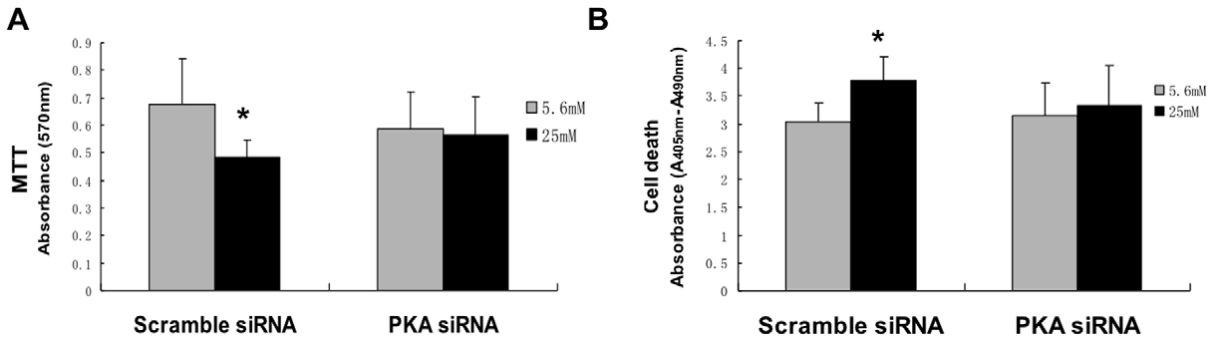
Inhibition of PKA by siRNA increased cell proliferation and decreases apoptosis in endothelial cells under high glucose treatment. Cells were transfected with siRNA and then treated with 5.6 mM glucose or 25 mM glucose for 72 hours: A: siRNA enhanced cell proliferation under high glucose conditions B: siRNA decreased apoptosis under high glucose conditions. *, p<0.05 compared with 5.6 mM condition. n = 6.

To determine whether rescuing G6PD activity improves phenotypic outcomes, the effects of siRNA inhibition of PKA was examined on cell growth and cell death. In previous published work, our laboratory has determined that increasing the activity of G6PD increases cell growth and decreases cell death [21], [22]. Thus we hypothesized that, at least in part, the PKA mediated decrease in G6PD played a central role in the high glucose mediated decrease in cell growth and increase in cell death. Figure 7 illustrates that high glucose decreased cell growth and enhanced apoptosis. Inhibition of PKA using the siRNA oligonucleotide ameliorated the inhibition of cell growth and ameliorated the high glucose mediated cell death.

### High glucose caused a decrease in G6PD activity, as well as an increase in NADPH oxidase activity

The reducing power of NADPH is used by many enzymes. Of particular interest is the NADPH oxidase (NOX) system, as this enzyme has been shown to be a main source of ROS in endothelial cells exposed to high glucose [24]–[26]. Thus, there appears to be a paradox in that studies have shown that high glucose causes a decrease in G6PD activity (and, as a result, a decrease in NADPH), yet many laboratories have shown that high glucose causes an increased activity of NOX which would seem to be require an increase in G6PD activity.

**Figure 8:**
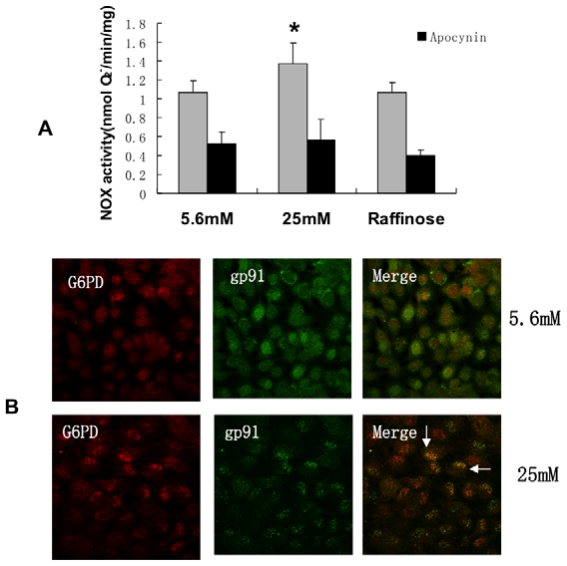
High glucose increased NOX activity as well as promoted colocalization of G6PD and NOX. Endothelial cells were treated for 72 hours with 5.6 mM or 25 mM glucose. A: NADPH oxidase activity was increased under high glucose conditions. Apocynin, an inhibitor of NOX, was used as an assay control. *, P<0.05 compared with 5.6 mM glucose and raffinose. See text for discussion. B: Colocalization of G6PD and gp91phox, a subunit of NADPH oxidase. BAECs grown on coverslips were stained with anti-G6PD (red, left panel) and anti-gp91phox (green, middle panel) antibodies. Colocalization of the fluorochromes results in a yellow colour (see arrows) which only occurred under high gluose conditions (right panel). n = 5.

To address this apparent paradox, we hypothesized that high glucose does indeed decrease G6PD (as we and others have shown) but that high glucose also stimulates colocalization of G6PD with NOX, thus possibly allowing adequate NADPH for optimal NOX activity despite an overall decrease in cellular NADPH due to decreased total cellular G6PD activity. Figure 1A showed that BAECs exposed to high glucose for 72 hours have decreased G6PD activity as compared to cells incubated with 5.6 mM glucose. Figure 8A shows that NADPH oxidase activity is increased by 25 mM glucose under the same conditions. Both the total lucigenin response (lucigenin is thought to primarily interact with superoxide) and the apocynin (an inhibitor of NADPH oxidase) inhibitable portion is shown in the figure. The results demonstrate that high glucose increases superoxide production from NADPH oxidase. Taken together, these results suggest that high glucose causes both an increase in NADPH oxidase and a decrease in G6PD activity.

### High glucose caused colocalization of G6PD and NADPH oxidase

To determinine if G6PD colocalizes with NOX, immunofluorescent staining was done. Figure 8B shows that there was no clear colocalization of G6PD (red) and the NOX subunit gp91 (green) in 5.6 mM glucose; however, 25 mM glucose led to colocalization as shown by the yellow color (overlapping of red and green) in many cells. These results suggest that high glucose causes colocalization of G6PD and NADPH oxidase which likely provides NADPH for NOX activity.

### Protein kinase A mediated colocalization of G6PD and NADPH oxidase

**Figure 9:**
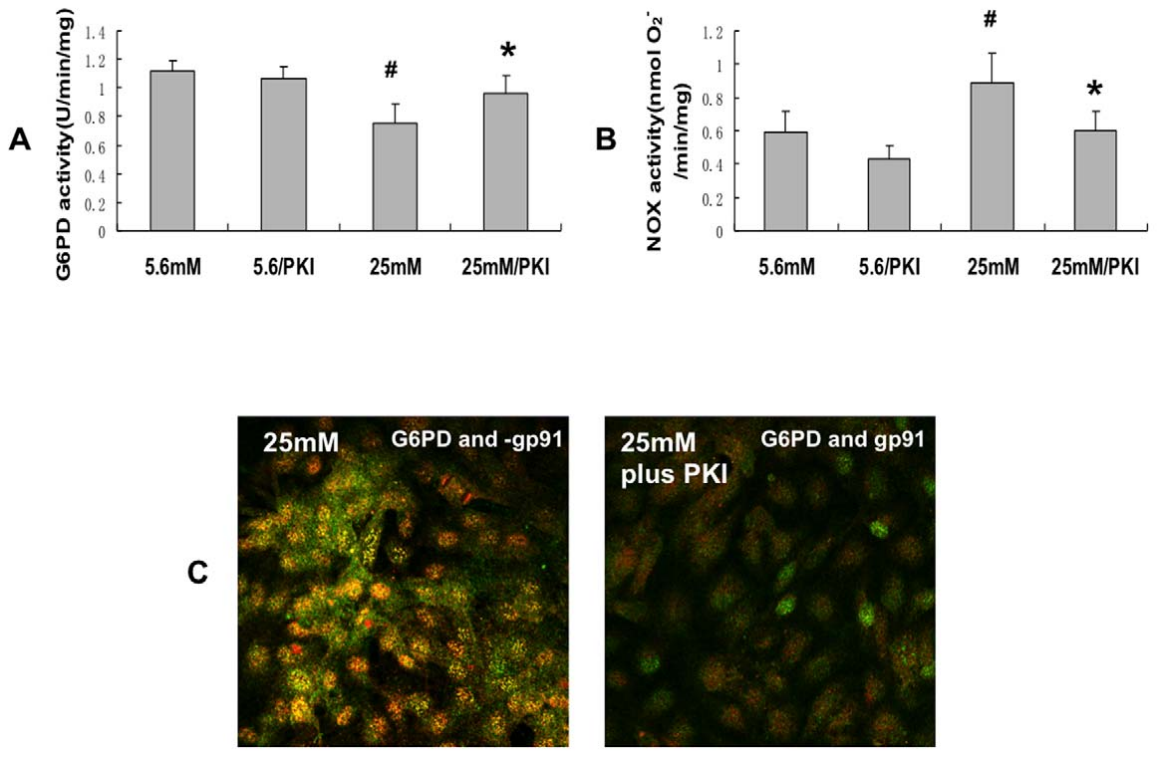
PKI (inhibitor of PKA) prevented the high glucose-induced decrease of G6PD activity, prevented the high glucose-mediated increase in NOX activity, and prevented colocalization of G6PD and gp91. A: Inhibition of PKA rescues the high glucose-induced decrease in G6PD activity. B: Inhibition of PKA prevents the high glucose-induced increase in NADPH oxidase activity. C: Left hand panel shows highly significant colocalization of G6PD and gp91 caused by high glucose and the right hand panel shows that inhibition of PKA by PKI prevents the colocalization by PKI. *, P<0.05 compared with 25 mM. #, P<0.05 compared with 5.6 mM. n = 5.

Since PKA mediates, at least in part, the high glucose-induced decrease in G6PD, we hypothesized that PKA may also mediate the high glucose induced colocalization of G6PD and NOX. Figure 9C illustrates that PKI inhibited the high glucose stimulated colocalization of G6PD and gp91 suggesting that increased PKA mediated the colocalization. Next it was determined whether increased PKA also regulated NOX activity. Figure 9A shows that PKI (the inhibitor of PKA) prevented the high glucose-induced decrease of G6PD activity as we have previously shown, and Figure 9B demonstrates that PKI decreased NADPH oxidase activity under high glucose conditions. These results suggest that PKA may mediate both the increase in NADPH oxidase activity and the decrease in G6PD activity caused by high glucose. Thus, in endothelial cells, high glucose stimulates a decrease in G6PD, and an increase in NOX. These changes in G6PD and NOX are mediated, at least in part, by increased PKA.

## Discussion

Inhibition of G6PD by high glucose has been previously observed by our laboratory and others. For example in cell culture models of endothelial cells and mesangial cells, G6PD is significantly inhibited by high glucose [27]. In animal models, decreased G6PD activity has been reported in liver [28], aorta [29], heart [30], [31], and Leydig cells [32]. In diabetic patients, decreased G6PD activity has been detected in percutaneous liver biopsies [32], mononuclear leukocytes [33], [34], and erythrocytes [35], [36]. These data reveal that high glucose-induced decrease in G6PD occurs in both diabetic models and diabetic patients and suggests that decreased G6PD may play a pathogenic role under high glucose conditions.

The importance of the high glucose mediated decrease in G6PD activity could only be inferred as previous studies did not enhance the activity of G6PD under high glucose conditions. The results reported in this paper, illustrate for the first time that increasing G6PD activity (either by overexpression or by inhibition of PKA) leads to improvement of redox status and redox enzymes and leads to enhanced cell growth and decreased cell death in endothelial cells. Thus the results here strongly support the hypothesis that decreased G6PD activity plays a central role in the high glucose mediated damage to endothelial cells. And that improving G6PD activity is potentially a valuable therapeutic goal.

The data reported here also suggest that inhibition of G6PD and the resulting decrease in NADPH likely mediate, at least in part, the high glucose-induced decreases in enzyme activities. As enzyme activity measurements are done in excess substrate conditions, the expected high glucose-stimulated decrease in NADPH cellular availability cannot be the only reason for decreased activities. Moreover although high glucose induced a decrease in the activities of catalase, GR and SOD, it didn't alter the protein expression of these enzymes. And overexpression of G6PD that rescued catalase activity and inhibition of PKA that led to rescuing of catalase, GR, and SOD activity did not result in any increase in protein expression of the redox enzymes. Hence, possibly by providing NADPH as a substrate or cofactor, G6PD was able to regulate the activities of other antioxidant enzymes. Other possible explanations are that overexpression of G6PD altered a signaling molecule that affected the activities of these enzymes or that altered redox status led to a change in a post-translational modification that affects specific activity of the enzyme(s).

**Figure 10:**
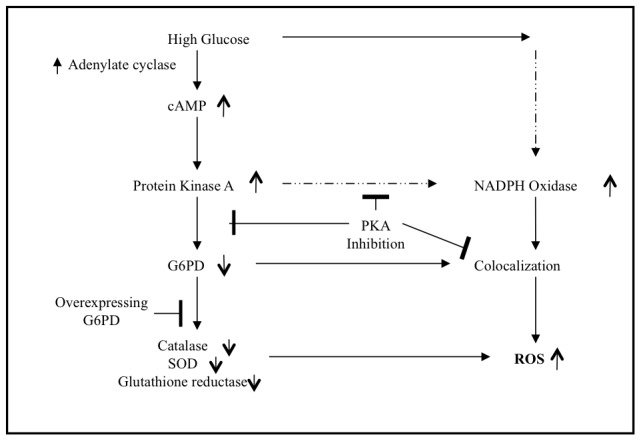
Proposed Model. High glucose stimulates cAMP which leads to activation of protein kinase A endothelial cells. PKA causes inhibition of G6PD and lowering of NADPH, which leads to decreased activities of catalase, SOD, and glutathione reductase. Activation of PKA also increases NOX activity and colocalization of NOX and G6PD. The overall effect of these changes is increased ROS level. Overexpression of G6PD will rescue the decrease in G6PD, NADPH, and other antioxidant enxymes. Also PKI and PKA specific siRNA (inhibitors of PKA) will also rescue the deleterious effects of high glucose on enzyme activities as well as on the colocalization of G6PD and gp91.

In this paper, the potentially central role for the high glucose mediated simulation of PKA is expanded from previous work. Our laboratory and others have previously reported that high glucose stimulates increased cAMP and protein kinase A in endothelial cells [9], [23], [37]. And we and others have previously shown that cAMP and cAMP-dependent protein kinase A regulates G6PD activity [27], [38], [39]. The data reported here illustrate that PKA also affects the activities of other critical antioxidant enzymes. Taken together, it is tempting to speculate that the mechanistic order is that high glucose stimulates an increase in PKA that subsequently inhibits G6PD activity and a resultant decrease in NADPH. And that the decreased NADPH causes a decrease in the enzyme activities (Figure 10). Although a direct effect of PKA on these enzymes or an indirect effect of PKA on another signaling pathway cannot be ruled out.

Researchers have demonstrated that high glucose activates NOX in endothelial cells, which plays an important role in endothelial injury and dysfunction [26], [40]. Since NOX activity is dependent on an adequate supply of NADPH, it would seem that G6PD activity should be increased to provide sufficient NADPH. Thus, there is an apparent paradox in that high glucose appears not only to decrease G6PD activity with a resulting decrease in NADPH, but also to increase NOX, which requires NADPH for ROS generation. Previous work from our laboratory first demonstrated (and since confirmed by others) that G6PD translocates inside the cell [20]. The results reported here show that high glucose stimulates colocalization of G6PD and NOX in endothelial cells. NOX has 7 known isoforms that are differentially expressed in specific cell types [41], [42]. Intracellular translocation of NOX and G6PD has been shown previously. The gp91phox subunit is expressed in BAECs and has been shown to be elevated under stress conditions [43] and the intracellular location well defined. The intracellular localization of gp91 (and the subsequent colocalization with G6PD) is consistent with what other laboratories have reported for the intracellular localization of gp91 [44]. It is possible that the close association of these two proteins allows sufficient NADPH to be delivered to NOX, even though total cellular G6PD activity is decreased. These results alone do not prove a mechanism but do provide an intriguing mechanistic model whereby targeting signaling molecules (e.g. inhibition of PKA) it is possible to improve redox balance by improving antioxidant enzyme function (increasing G6PD activity) and decreasing oxidant production (lowering NOX activity).

There are studies that have evaluated the effects of cAMP and PKA on NADPH oxidase. Some studies on NOX1 have shown that increased PKA leads to inhibition of activity [45]–[47]. Muzaffar and others reported that PKA regulated the expression of gp91 in arterial endothelial cells (49). Another study in granulocytes from type 2 diabetic patients showed that granulocytes from non-diabetic patients have decreased reactive oxygen species production (which was primarily derived from NADPH oxidase) following stimulation with cAMP, but granulocytes from diabetic patients had increased ROS production after stimulation of PKA [48]. Thus, it is quite possible that diabetes alters the metabolic signaling pathways that regulate NADPH oxidase. It is also possible that the isoforms of NOX respond differently to increased cAMP and PKA. Indeed, considering the variable effects of high glucose on PKA and the ubiquitous role that PKA plays in many cell types and on many cell activities, much more will need to be understood about PKA and its regulation of G6PD and NADPH oxidase, in order to develop treatments that specifically target the PKA in endothelial cells under high glucose conditions to improve overall function and survival.

Lastly, many of the observed changes in redox enzymes are relatively small yet statistically significant. These results raise the question as to the physiologic importance of small changes in enzyme activity. In previous studies we have shown that similarly small changes in G6PD can lead to significant changes in cell phenotypes such as cell growth, cell death, and angiogenesis [21], [22], [49], [50]. In addition, in the information reported in this paper, restoring these relatively small changes in metabolic enzymes (either by overexpressing G6PD or by inhibition of PKA) led to restoration in ROS balance, enhanced cell growth, and decreased cell death. Thus although these enzymatic changes are relatively small, they are physiologically relevant.

In conclusion, the data reported here provide new insights into the mechanisms underlying the deleterious effects of high glucose on endothelial cells by illustrating the likely central pathophysiologic role for decreased G6PD activity and increased PKA in endothelial cells. Future studies using therapeutic approaches that increase G6PD and/or inhibit PKA in animal models of diabetes should provide further insights into the development of new possible treatments.

## Materials and Methods

### Cell Culture

Bovine aortic endothelial cells (BAEC) were freshly isolated by scraping the luminal side of a calf aorta from Dr. C. Rask-Madsen (Joslin Diabetes Center, Boston), cultured and identified as previously described [51]. Cells between passage 3 and 6 were used. The cells were grown in DMEM with 10% calf serum. For the adenoviral infection studies the cells were allowed to reach 90% confluent then infection was performed with pAd-G6PD (MOI: 5) or empty vector. After 24 hours, medium was switched to DMEM with 1% serum plus 5.6 mM glucose, 25 mM glucose or 25 mM raffinose for 72 hours. For the inhibition studies using the pharmacologic PKA activity, the specific cell-permeable PKA inhibitor 14–22 amide (PKI) (10 µmol/l) was added to the medium for the last 24 hours. Cells were harvested for further experiments.

### Construction of Adenoviral-hG6PD expression vector

Human G6PD cDNA was excised from pCMV6_XL5-G6PD by EcoR I and Xba I digestion and inserted into a shuttle vector, pHIHG-Ad2. The resulting plasmid was digested with PacI and MfeI; the fragment containing G6PD cDNA was used to transform Escherichia coli BJ5183 together with a ClaI-linearized adenovirus vector, pAd-hGM-CSF. Homologous recombination of the two DNA fragments in BJ5183 produced a new adenoviral vector, pAd-G6PD, in which hGMCSF in the original vector was replaced by G6PD. pAd-G6PD was extracted from BJ5183 and transferred to E. coli XL-10 for large scale plasmid preparation. The sequence of pAd-G6PD was confirmed by sequencing. Expression of G6PD was confirmed by infection of HEK-293 cells followed by Western blotting. The titer of purified adenovirus was determined (Adeno-X™ Rapid Titer Kit, Clontech) according to manufacture's instructions. Empty vector was used for control experiments.

### Duplex siRNA Targeting Constructs and Transfection

Small interfering RNA duplex oligonucleotides were purchased from Dharmacon, Inc. (Lafayette, CO). The sequence of the siRNA duplex construct targeting PKA was 5′-GAGUAAAGGCUACAACAAA-dTdT-3′, corresponding to bases 637–655 from the open reading frame of the bovine PKA catalytic subunit mRNA (GenBankTM accession number NM_174584). The duplex siRNA used as a scramble siRNA control was 5′-GCCCGCUUUGUAGCAUUCG-dTdT-3′. In preliminary experiments, we optimized the conditions for the efficient transfection of BAEC using siRNA. We found that optimal conditions for siRNA knockdown involved transfecting BAEC at 70–80% confluency maintained in DMEM/10% calf serum. For the transfections with siRNA (5 nM) LipofectAMINE 2000 (0.075% v/v) was used following protocols provided by the manufacturer. Fresh medium was added 5 hours post-transfection, After 24 hours, the medium was switched to DMEM with 1% calf serum plus 5.6 mM glucose or 25 mM glucose for 72 hours.

### Measurement of G6PD, Catalase, Glutathione Reductase (GR), and Superoxide Dismutase (SOD) activity

G6PD activity was measured as described previously [23]. Activity of catalase, SOD, and GR was measured by spectrophotometric methods according to the manufacture's instructions (Cayman, MI).

### Measurement of NADPH, GSH/GSSG and Thiobarbituric Reactive Substances (TBARS)

NADPH was measured by a colorimetric method according to the manufacture's instructions (Bioassay System, CA). GSH/GSSG was measured by a spectrophotometric method according to the manufacturer's instructions (Cayman, MI). TBARS level was measured by a fluorometric method according to the manufacture's instructions (ZeptoMetrix Corporation, NY).

### Measurement of ROS accumulation

ROS production was measured with the dye CM-dihydro-dichlorofluorescein diacetate (H_2_DCFDA) (Invitrogen). Fluorescence was determined in a microplate fluorometer (Victor^2^ fluorometer, PerkinElmer).

### Measurement of Cell Proliferation and Apoptosis

Cell proliferation was measured by spectrophotometric methods using the MTT cell proliferation assay kit according to the manufacture's instructions (Cayman, MI). Apoptosis was measured by a photometric enzyme-immunoassay using the cell death detection ELISA kit according to the manufacture's instruction (Roche Diagnostics, IN).

### Measurement of NADPH Oxidase Activity

NOX activity assay was measured as described previously [52].

### Immunofluorescence labeling for confocal microscope

Double labeling was performed on BAECs grown on coverslips with rabbit anti-G6PD (Abcam) and mouse anti-gp91 primary antibodies.

### Statistical analysis

Data are expressed as means±SD. The significance of the differences in mean values among different groups was evaluated using one-way ANOVA and a post hoc analysis using the Tukey test. P<0.05 was considered statistically significant.

## Supporting Information

Figure S1 Overexpression of G6PD does not affect the protein expression of catalase in BAECs.
(DOC)

Figure S2 Overexpression of G6PD does not affect the protein expression of glutathione reductase in BAECs.
(DOC)

Figure S3 Overexpression of G6PD does not affect the protein expression of SOD in BAECs.
(DOC)
