# Supplementary figures and images for: Increasing Glucose 6-Phosphate Dehydrogenase Activity Restores Redox Balance in Vascular Endothelial Cells Exposed to High Glucose
Source: PLoS One. 2012 Nov 19;7(11):e49128. doi: 10.1371/journal.pone.0049128 (PMC3501497; doi:10.1371/journal.pone.0049128)

**Supplementary Data**


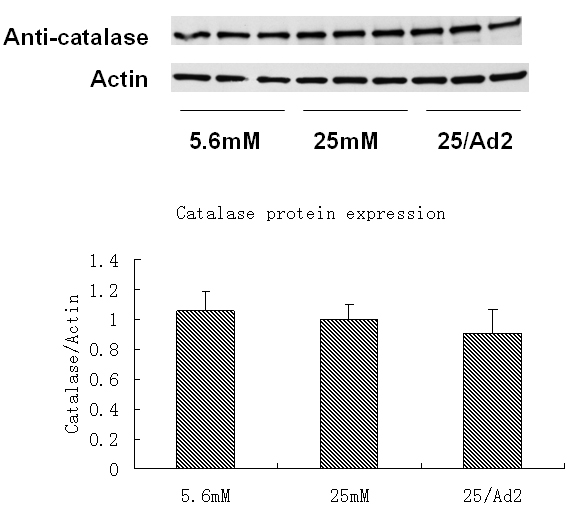


Figure S1.

Supplement: Figure S1 — Overexpression of G6PD does not affect the protein expression of catalase in BAECs. (DOC) [file pone.0049128.s001.doc]

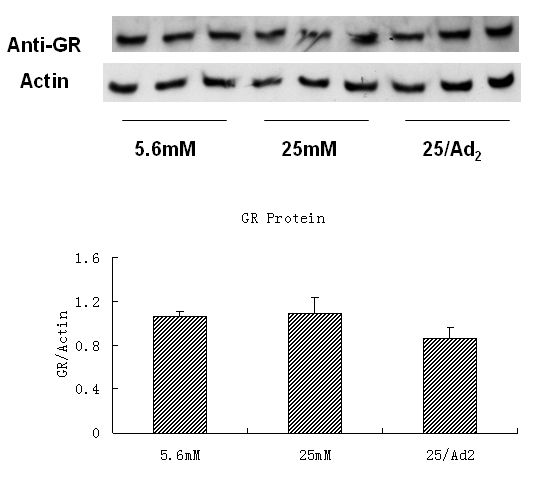


Figure S2.

Supplement: Figure S2 — Overexpression of G6PD does not affect the protein expression of glutathione reductase in BAECs. (DOC) [file pone.0049128.s002.doc]

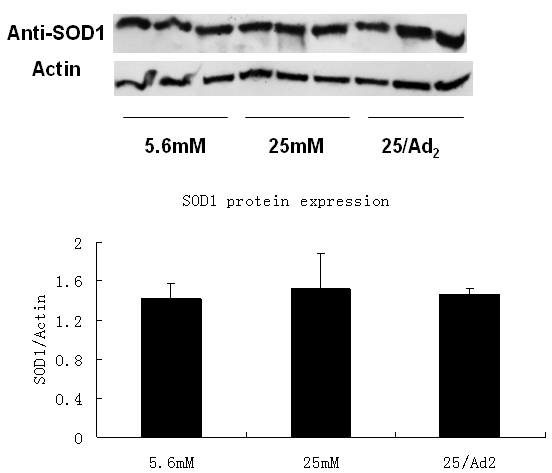


Figure S3.

Supplement: Figure S3 — Overexpression of G6PD does not affect the protein expression of SOD in BAECs. (DOC) [file pone.0049128.s003.doc]
